# Dental Society of the State of Maryland

**Published:** 1868-12

**Authors:** S. M. Field, Jas. B. Bean

**Affiliations:** Rec. Sec.; President.


### CORRESPONDENCE.

### ARTICLE IV.

### *Dental Society of the State of Maryland.*

The regular monthly meeting was held Oct. 29th, at the rooms of the Association in Baltimore. The President, Dr. J. B. Bean, in the Chair.

After the transaction of the usual formal business on the meeting of the Society, the discussion of the 5th of Dr. Arthur's propositions was called for, but, on motion, was adjourned over until the December meeting. The propositions are as follows:

1st.—That caries will attack the proximate surfaces of all the teeth, except the inferior incisors, of the great majority of persons in the United States, at the present day.

When caries of the superior incisors occurs on the proximate surfaces previously to the twelfth year, its occurrence, sooner or later, on the same surfaces of all the teeth, except the inferior incisors is nearly certain. In the greater number of such cases the caries will show itself before the twentieth year.

This predisposition to dental caries is greater in the female sex.

2d.—That caries is not liable to occur, at the points indicated, unless the teeth are in contact.

3d.—That an artificial, permanent separation of the teeth will arrest superficial caries, or prevent its occurrence if the attack has not actually begun.

4th.—That it is a popular fallacy to suppose that caries necessarily follows the removal of the enamel.

5th.—That the most efficient means of preserving the teeth is to anticipate the attack of caries by separating them, when it is ascertained that caries is likely to occur on the proximate surfaces.

Dr. Arthur then said:—

Mr. President:

The propositions offered by me, at the January meeting of this society, as the members present are aware, came up for discussion, as agreed upon, at the meeting held in June. At the termination of the discussion on that occasion (if the little then said deserves the name,) I requested that no vote should be taken upon the propositions in question, but that action should be deferred until the next regular meeting, so that further opportunity might be afforded to any one, who might undertake to do so, to show the unsoundness of the views embodied in them if they could not bear closer scrutiny. At the meeting referred to the propositions were taken up, *seriatim*, slightly modified, and with the exception of the last, unanimously endorsed. Several members present, however, expressed some reluctance to give their unqualified approval of the last proposition, and desired that an opportunity might be afforded for its further discussion.

I proposed, therefore, that the matter should be deferred until the present meeting. I did this, not because I entertained any doubt about the entire truth of the proposition referred to, which, indeed, is an inevitable sequence of those which precede it, but in order that the plan of practice I propose, should be subjected to the most searching examination. It has now been determined still further to postpone the discussion, until the December meeting, and for the reason stated this action meets with my entire approval.

I beg to say again, Mr. President, at this time as I have before said, that the method of treating dental caries, indicated in the propositions before the Association, is not a crude or immature idea, but is based on the deliberate conclusion arrived at as one of the results of a long series of careful observations, and tested fully in actual practice for a number of years.

I have urged these views earnestly and persistently because I am deeply impressed with their great importance. It must be evident that I have no personal object to accomplish in pressing this matter. I would not, I assure you, walk across the street to induce any one to adopt opinions of mine merely as a matter of self-gratification. But the method of practice I propose, if it be correct, ought to engage the earnest attention of every dentist who has any desire to practice his art for the good of the public, as it is claimed for it that a great amount of suffering will be prevented by its general adoption. If it is erroneous its fallacy should be exposed. If it is erroneous I am doing great injury, not only in my own practice, in which for a number of years I have been pushing it vigorously, but I am misleading many men, in the dental profession, who come within my influence.

But although the views of practice referred to have been before the dental profession for some three years, there has, as yet, been no attempt made to controvert it, at all worthy of notice.

There is but little doubt, however, that what I mean is not understood by members of the dental profession so far as my views may have attracted their attention. One of my old professional friends declares to me that he can account for my views only on the ground of "lunacy" A sapient critic who occupied the position of chairman of the "Committee of Dental Literature," of the "American Dental Association" at its last meeting, pronounces the views presented in my little work on "Dental Caries," "*peculiar* and *antiquated*." Certainly a very remarkable and original combination! How my peculiarities of opinion can be identical with the views of my ancient *confreres* in the dental profession I am not able to understand. I am at the same time quite as unable to understand how any man of ordinary intelligence, who has practiced dentistry a single year, can fail to comprehend, on reading it, what the little treatise referred to, does mean. It was not written, it is true, for the dental profession, but, for that very reason, was made so simple in its statements as to reach the comprehension of persons who had not given the subject any attention at all. I have conversed with no intelligent person, outside of the dental profession, who has hesitated to accept the views referred to as beyond dispute. If this matter could be comprehended by persons not acquainted with dentistry it seems unaccountable that those who are informed on the general subject should be the last to understand the full scope of the ideas advanced.

Shortly after the publication of the monograph referred to, it attracted the attention of Prof. Noel of the Baltimore College of Dental Surgery, it was reviewed by him, very fully and favorably, in the American Journal of Dental Science, Nos. 3, 4, 5, 6, 7.

It has been sneeringly said that he was, in this case, writing about a subject with which he was not well acquainted; but

I think any well informed and candid dentist, who reads the whole of Prof. Noel's article, will admit that he has furnished one of the ablest *resumes* of the various theories of dental caries that has yet appeared. It would be as well for the dental profession, if those who undertake to enlighten it by their contributions to the "Journals" would make themselves as capable of discussing the principles upon which it is based as Prof. Noel.

The general impression seems to be that I have advised the indiscriminate filing of sound teeth as a preventive of caries; or a separation, by means of a file, of all the teeth in contact, of every young person who comes under my professional care. I have advised no such thing, and the whole tenor of the argument of the little book referred to, is simply to prove: that caries of the teeth is always external in its origin; that it is caused by the action of a decomposing agent which exerts its influence slowly, and requires to be kept for some time in contact with the particular portion of a tooth which decays; that experience, dating long anterior to my own, and in that respect quite "antiquated," has established the fact that an artificial, permanent separation of teeth, will arrest superficial caries more effectually than the most perfect filling; that, when it becomes apparent that decay will occur where the teeth are in contact, it is the best practice to make the separation before the progress of the caries has rendered this method of treatment impossible; that, as a general rule, if caries attacks the proximate surfaces of the incisor teeth of a child, previous to the twelfth year, it is reasonably certain to attack the proximate surfaces of all the teeth, except the inferior incisors, before the twentieth year; and that the sooner the necessary separations are made, the greater will be the advantage gained.

There is certainly no ambiguity about this, and I am so well convinced of the truth of my views that I am ready to meet any gentleman or gentlemen in the dental profession, in or out of this Association, or in or out of this city, at the regular meeting of the Association, in December, or at any other time, and to discuss the views referred to, in all their aspects.

I have said that I am interested in the matter so far only as it is one of public interest, and I would call the attention of the members present to the fact that the preservation of the teeth, especially the bicuspids, by filling, after the caries has progressed so far as to become apparent upon a mere visual examination, is very difficult. I think you will bear me out in the statement that a great proportion of the 10.000 dentists, the estimated number now practicing in this country, do not possess the skill necessary to accomplish this object, and if they did, the great mass of the public would not be willing, and a large part would be unable, to pay a sum which would enable an operator to spend the time and nice care which are absolutely necessary to insure a good result. The same may, indeed, be said, but not to the same extent, of cavities occurring on the proximate surfaces of the molar teeth.

Vast numbers of operations, in the way of filling, at these positions, you will bear me witness, are entirely worthless, either because the operator is incapable of performing them well, or cannot command sufficient compensation to enable him to employ the best material, and to devote the necessary time and care to their performance.

Another feature of the matter, too, and one which deserves the earnest attention of every humane man is, that, after the caries has progressed so as to render filling necessary, the operations become, in the great majority of cases, extremely painful, so much so indeed, that many persons are entirely unable to endure the suffering occasioned by them, while by the method of treatment, I propose, this suffering is almost entirely avoided.

I strongly urged the advantages of this method of treatment on the grounds: 1st. That caries can be more certainly arrested by a thorough permanent separation than by filling. 2nd. That the operation is simpler and within the reach of the ability of any practitioner who will do his work faithfully. 3rd. That it is not attended with pain. 4th. It is less expensive, and thus within the reach of many persons who would, otherwise, be compelled to permit their children to lose their teeth.

It must be admitted that the advantages claimed for this method of practice are great. Do they exist? If I am correct in my views, an immense amount of unnecessary pain is daily inflicted, and serious injury done. If I am wrong it is certainly desirable that my errors should be exposed, and no one has a greater interest than myself, in reaching correct views upon every point of importance relating to my profession.

S. M. Field, *Rec. Sec.* — Jas. B. Bean, *President.*

